# How are falls and fear of falling associated with objectively measured physical activity in a cohort of community-dwelling older men?

**DOI:** 10.1186/1471-2318-14-114

**Published:** 2014-10-27

**Authors:** Barbara J Jefferis, Steve Iliffe, Denise Kendrick, Ngaire Kerse, Stewart Trost, Lucy T Lennon, Sarah Ash, Claudio Sartini, Richard W Morris, S Goya Wannamethee, Peter H Whincup

**Affiliations:** Department of Primary Care & Population Health, UCL Medical School, Rowland Hill Street, London, NW3 2PF UK; Division of Primary Care, School of Medicine, University of Nottingham, Nottingham, UK; Department of General Practice and Primary Health Care, School of Population Health, University of Auckland, Auckland, New Zealand; School of Human Studies, University of Queensland, Queensland, Australia; Population Health Research Institute, St George’s, University of London, Cranmer Terrace, SW17 0RE London, UK

**Keywords:** Falls, Fear of falls, Physical activity, Accelerometer, Older adults

## Abstract

**Background:**

Falls affect approximately one third of community-dwelling older adults each year and have serious health and social consequences. Fear of falling (FOF) (lack of confidence in maintaining balance during normal activities) affects many older adults, irrespective of whether they have actually experienced falls. Both falls and fear of falls may result in restrictions of physical activity, which in turn have health consequences. To date the relation between (i) falls and (ii) fear of falling with physical activity have not been investigated using objectively measured activity data which permits examination of different intensities of activity and sedentary behaviour.

**Methods:**

Cross-sectional study of 1680 men aged 71–92 years recruited from primary care practices who were part of an on-going population-based cohort. Men reported falls history in previous 12 months, FOF, health status and demographic characteristics. Men wore a GT3x accelerometer over the hip for 7 days.

**Results:**

Among the 12% of men who had recurrent falls, daily activity levels were lower than among non-fallers; 942 (95% CI 503, 1381) fewer steps/day, 12(95% CI 2, 22) minutes less in light activity, 10(95% CI 5, 15) minutes less in moderate to vigorous PA [MVPA] and 22(95% CI 9, 35) minutes more in sedentary behaviour. 16% (n = 254) of men reported FOF, of whom 52% (n = 133) had fallen in the past year. Physical activity deficits were even greater in the men who reported that they were fearful of falling than in men who had fallen. Men who were fearful of falling took 1766(95% CI 1391, 2142) fewer steps/day than men who were not fearful, and spent 27(95% CI 18, 36) minutes less in light PA, 18(95% CI 13, 22) minutes less in MVPA, and 45(95% CI 34, 56) minutes more in sedentary behaviour. The significant differences in activity levels between (i) fallers and non-fallers and (ii) men who were fearful of falling or not fearful, were mediated by similar variables; lower exercise self-efficacy, fewer excursions from home and more mobility difficulties.

**Conclusions:**

Falls and in particular fear of falling are important barriers to older people gaining health benefits of walking and MVPA. Future studies should assess the longitudinal associations between falls and physical activity.

## Background

Physical activity (PA) levels in older people are low and decline with increasing age [1]. In the UK only 9% of men and 6% of women over 75 years [1] report achieving recommended levels of moderate to vigorous physical activity (MVPA) of 150 minutes per week [2, 3]. These low PA levels have deleterious effects on a wide range of health outcomes [4].

Falls are very common in community-dwelling older adults; approximately one third report falling in the past 12 months [5]. Falls have serious physical and psychological consequences for individuals, for society, and for health services due to the high cost of inpatient admissions and long term care [6]. Fallers have lower levels of self-reported PA [7], perhaps due to mobility limitations after an injury or avoiding activities because of fear of falling. The lower activity levels may in turn decrease strength and balance and initiate a downward cycle towards losing independence and entering long-term care.

Fear of falling (FOF) has been variously defined including concern that normal activities cannot be performed without falling, lack of confidence in maintaining balance during normal activities and being frightened of falling [8]. FOF affects 20-50% of older adults [9–11] and may be a rational psychological response to previous falls, but is also reported by people who have not fallen [12]. FOF is associated with an increased falls risk, functional restrictions, lower quality of life and low PA levels [11–16]. Whilst FOF may result in taking extra care during activities, possibly preventing falls, the reduction in activity may also lead to deconditioning and loss of muscle strength [17].

Much research about PA and falls focuses on exercise interventions to reduce onset of falls in the community [18]. To date, less research focuses on the related questions of how PA levels may be restricted after experiencing a fall, potentially because FOF may result in activities being curtailed. Indeed, previous studies find that self-reported PA levels are lower after a fall and in people who fear falling [7, 11–15, 19]. However, to our knowledge, no studies have examined how PA levels and sedentary behaviour, measured using accelerometers, vary according to history of falls and FOF. Accelerometers permit investigation of which intensities of activity are affected, for example it could be that after a fall or in people who are fearful of falling, MVPA may be reduced and replaced with light intensity activity, or alternatively, that light intensity activities are also reduced and then sedentary time is increased too. Given the high prevalence of falls and of FOF in older adults, it is important to understand how they are associated with different intensities of physical activity.

We therefore aimed to investigate associations between (i) history of falls and (ii) FOF with objectively measured PA, (step counts and daily minutes in sedentary, light and MVPA), and what factors may mediate any associations, using a large sample of independently mobile, community-dwelling older men.

## Methods

### Sample

**Figure 1:**
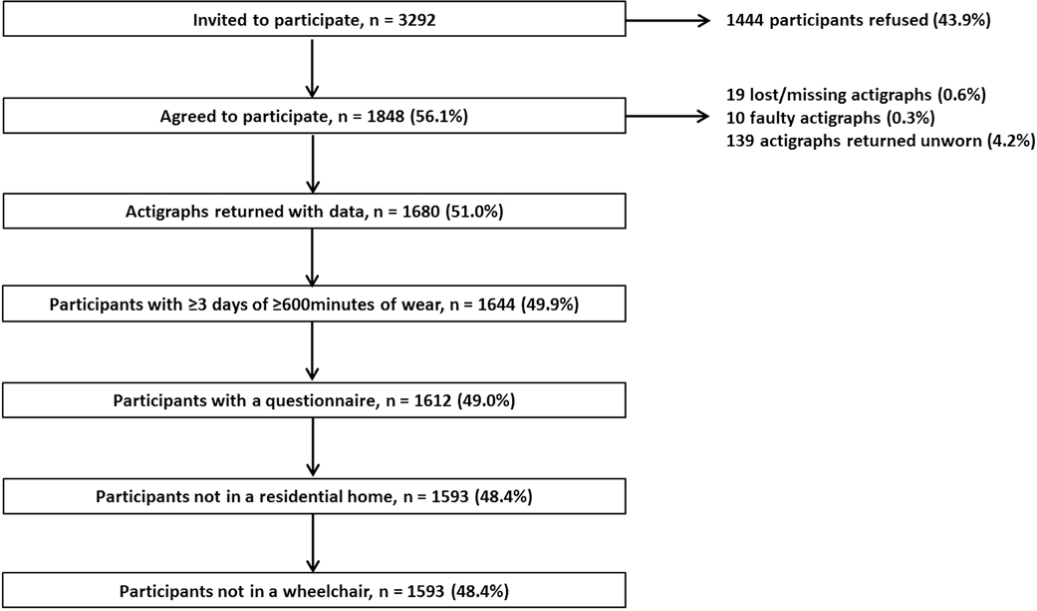
**Flow chart identifying the sample of men.**

The British Regional Heart Study is a prospective cohort of 7735 men recruited from a single Primary care centre in 24 British towns in 1978–80 (age 40–59 years). In 2010–2012, 3292 survivors were invited by post to participate in a study of objectively measured PA (Figure 1). The National Research Ethics Service (NRES) Committee for London provided ethical approval. All men provided informed written consent to the investigation, which was performed in accordance with the Declaration of Helsinki.

### Measures

#### Accelerometer data

Participants wore a GT3X accelerometer (Actigraph, Pensacola, Florida) over the hip for 7 days, during waking hours, removing it for bathing. Uniaxial data were analysed in 60 second epochs. Non-wear time was identified and excluded using a commonly used, freely available R package [20]. Men with ≥3 days of ≥600 minutes of wear time were included in analyses. Extreme data points (step counts <100 or >20,000/day) were verified against log diaries. The number of minutes spent in PA of different intensity levels was categorised using standard counts per minute (cpm)-based intensity threshold values of <100 for sedentary behaviour, 100–1040 for light activity and >1040 for MVPA (a cut-point for older adults) [21–23].

#### Questionnaire data

Men completed a log diary (detailing accelerometer wear times), and questionnaire including the following questions: “Have you had a fall in the past 12 months?” [yes/no] and “if yes, how many falls have you had in the past 12 months?” This question has high specificity and acceptable sensitivity for detecting falls in the previous 12 months [24]. “At the present time are you afraid that you may fall over?” [very fearful and somewhat fearful were compared to not fearful]. Participants completed the Self-Efficacy for Exercise scale [25] and the Expected Outcomes for Habitual Exercise scale [25]. Men scoring two or more on the 4 item Geriatric Depression scale (GDS) were classified as having depression [26], Men reported whether they had problems getting about outdoors and on how many days in the previous week they left the house. Men answered five standard questions about mobility, self-care, usual activities, pain or discomfort and anxiety or depression from which the EuroQol EQ-5D health related quality of life score was calculated [27], this score was transformed into a z-score for analyses. Social position was based on longest held occupation reported in previous surveys [manual/ non-manual].

#### Statistical methods

Associations between confounding and potential mediator variables and falls history were examined. Summary measures of confounding and potential mediator variables were calculated according to falls history. Linear regression models were used to examine differences in continuous variables across the three groups of non-fallers, single fallers and recurrent fallers, differences in categorical variables were tested using Chi Squared tests. The same approach was taken for FOF. Associations between each of the potential mediators and the PA outcome variables were evaluated using random effects linear regression models.

Daily steps and minutes of sedentary and light activity were approximately normally distributed. MVPA had an excess of zero counts so was transformed (using log or square root transformations), but patterns of results did not differ from analyses using untransformed data, so the latter are presented for ease of interpretation. Random effects linear regression models were used to assess the associations between falls and each of four different PA outcome measures (daily steps, minutes of sedentary, light and MVPA), accounting for clustering with PA outcome (range 3–8 days of accelerometer data) at level 1 and person at level 2. The xtmixed command in Stata was used, specifying a random intercept and identity correlation structure. The mean difference in each of the four PA outcomes was compared between 3 groups; (i) men had not fallen in the past 12 months (baseline), (ii) men who had fallen once and (iii) men with recurrent (> = 2) falls. Models were adjusted for confounders; age, day order, month, wear time (minutes/day) and town of residence. Mediators between falls history and PA level were chosen if they had been reported to be associated with PA levels and with falls or FOF in other studies, and were also related to falls, FOF and PA levels in our study. These mediators were added one by one, to evaluate the role of each; exercise self-efficacy, exercise outcome expectations (both analysed as standard deviation scores), mobility problems, number of days leaving the house, depression, health-related quality of life score, and FOF. A final model included all potential mediators to evaluate whether associations between falls and PA were fully mediated. Complete case analysis was used.

The same modelling strategy was used for FOF; men who were currently “very” and “somewhat” fearful were grouped together and compared to men who were not fearful of falling. Falls history was included as a covariate in models of FOF. Interactions between FOF and fall history on step count, minutes of sedentary, light and MVPA were investigated, and change in model fit was evaluated using a likelihood ratio test (LRT). As a sensitivity analysis, we investigated the impact of using lower cut points of 25 cpm [28] and 50 cpm [29] rather than 100 cpm to define sedentary behaviour. Analyses were conducted using Stata version 12 [30].

## Results

1680/3292 (51%) of men invited agreed to participate and had accelerometer data (Figure 1). The sample was restricted to 1593/1680 (95%) independently mobile community-dwelling men with > =600 minutes wear time on 3–8 days, with mean age 78.3 years (range 71–93).

### Falls history and physical activity

21% (328/1568) of men with falls data reported at least one fall in the previous 12 months; 9% (n = 143) had one fall and 12% (n = 185) had recurrent falls (range 2–25). Compared to men who did not fall, single and recurrent fallers were significantly older, more fearful of falling, had more mobility difficulties outdoors, left the house less often, had higher prevalence of depression, lower exercise self-efficacy (they were less confident about being able to exercise in the face of difficulties), lower exercise outcomes expectations (expectations about the benefits of exercise for them) and lower quality of life. Men who did not fall took significantly more steps per day and spent more time in light and in moderate or vigorous activity than recurrent fallers, (Table 1) at the time of the survey. Men in manual social class were less likely to have a single fall and more likely to have recurrent falls than men in non-manual social class. Each one of the mediator variables was associated with the number of minutes in sedentary, light and MVPA and steps per day (data not presented).

**Table 1:** **Characteristics of men according to falls history (n = 1568), and fear of falling (n = 1577)**

	No falls	1 fall	≥2 falls	Total	p value	Not fearful of falling	Fearful of falling	Total	P value
%(n)	79.1(1240)	9.1(143)	11.8(185)	100(1568)		83.9(1323)	16.1(254)	100(1577)	
Age, years (mean, SD)	77.9(4.4)	79.8(5.0)	79.5(5.0)	78.3(4.6)	<0.001	77.9(4.4)	80.4(5.0)	78.3(4.6)	<0.001
Region, %(n)					0.089				0.045
South	34.1(423)	35.0(50)	28.6(53)	33.5(526)		33.6(444)	33.5(85)	33.5(529)	
Midlands	14.8(184)	15.4(22)	11.4(21)	14.5(227)		14.0(185)	16.5(42)	14.4(227)	
North	41.2(511)	35.7(51)	51.4(95)	41.9(657)		42.2(558)	42.5(108)	42.2(666)	
Scotland	9.8(122)	14.0(20)	8.6(16)	10.1(158)		10.3(136)	7.5(19)	9.8(155)	
Manual social class, %(n)	46.4(516)	33.9(42)	55.1(87)	46.3(645)	0.002	45.5(537)	52.0(116)	46.5(653)	0.074
Fearful of falling, %(n)	9.5(117)	26.1(37)	52.5(96)	16.1(250)	<0.001	-	-	-	
One fall in past 12 months, %(n)	-	-	-	-		8.0(105)	14.8(37)	9.1(142)	<0.001
≥2 Falls in past 12 months, %(n)	-	-	-	-		6.7(87)	38.4(96)	11.8(183)	
Mobility limitations, %(n)	9.1(112)	17.5(25)	44.3(82)	14.0(219)	<0.001	7.1(92)	51.0(127)	14.1(219)	<0.001
N days leave the house/past week, mean (SD)	6.3(1.5)	6.2(1.7)	5.0(2.3)	6.1(1.7)	<0.001	6.3(1.4)	4.8(2.4)	6.1(1.7)	<0.001
Depressed (>=2, Geriatric Depression Score), % (n)	19.9(245)	24.5(35)	43.7(80)	23.1(360)	<0.001	17.7(233)	50.6(127)	23.0(360)	<0.001
Exercise self-efficacy, (z-score), mean (SD)	0.1(0.9)	-0.8(1.0)	-0.6(1.0)	0.0(1.0)	<0.001	0.2(0.9)	-0.9(0.9)	0.0(1.0)	<0.001
Exercise outcome expectations, (z-score), mean (SD)	0.6(0.9)	0.0(1.0)	-0.4(1.1)	0.0(1.0)	<0.001	0.1(0.9)	-0.5(1.1)	0.0(1.0)	<0.001
Quality of life (EQ-5D), z-score, mean (SD)	0.2(0.8)	-0.3(1.1)	-0.4(1.1)	0.0(1.0)	<0.001	0.2(0.7)	-1.0(1.2)	0.0(1.0)	<0.001
Steps/day, mean (95% CI)^1^	4968(4826, 5110)	4711(4289, 5134)	3417(3101, 3733)	4767(4642, 4892)		5147(5008,5285)	2702(2491,2912)	4764(4642,4885)	
Sedentary (minutes/day), mean (95% CI)^1^	621(616,625)	634(623, 546)	643(631,654)	624(621,628)		618(614,622)	664(655,673)	625(621,629)	
Light PA (minutes/day), mean (95% CI)^1^	199(195,201)	187(177,196)	174(165,184)	195(192,198)		201(198,204)	158(151,166)	195(192,198)	
MVPA (minutes/day), mean (95% CI)^1^	40(39,42)	37(32,41)	24(21,27)	38(37,40)		42(40,44)	17(15,19)	38(37,40)	

^1^adjusted for minutes of accelerometer wear time, region of residence, day order, age and season of wear.

Results from regression models indicate that single fallers took a similar number of steps/day, and spent a similar number of minutes in sedentary, light and MVPA compared to non-fallers (Table 2a, Model 1). However, recurrent fallers took significantly fewer steps/day than non-fallers (adjusted mean difference -942 (95% CI -503, -1381) and spent more minutes sedentary 22(95%CI 9, 35), and less in light PA -12(95% CI -2, -22) MVPA -10(95% CI -5,-15), [Table 2b, Model 1]). These differences were abolished after adjustment for quality of life (Model 8), exercise self-efficacy (Model 3), fear of falling (Model 6) and mobility limitations (Model 5). The differences in step count were attenuated but remained significant after adjustment for leaving the house on fewer days (Model 4), lower exercise outcome expectations (Model 2) and depression (Model 7). The differences in sedentary and light time were fully mediated by adjustment for each of the mediators. The differences in MVPA were partially mediated on adjustment for exercise outcome expectations (Model 2), leaving the house (Model 4) and depression (Model 7), and fully mediated with each of the other single adjustments and when all covariates were included (Model 9).

**Table 2:** **(a and b) Adjusted mean difference (95% CI) in PA level between non-fallers and (a) one fall (b) ≥2 falls (n = 1398)** ^**1**^

(a)No fall vs one fall in past 12 months	Steps/day	Sedentary (minutes/day)	Light (minutes/day)	MVPA (minutes/day)
model 1 = falls in past 12 months	-14(-482,454)	10(-4,24)	-9(-20,1)	-1(-7,5)
model 2 = model 1+ exercise outcome expectations	39(-402,480)	9(-4,22)	-8(-19,2)	0(-6,5)
model 3 = model 1+ exercise self-efficacy	166(-264,597)	6(-7,19)	-7(-17,4)	1(-4,6)
model 4 = model 1+ number of days leave the house	6(-443,455)	10(-4,23)	-9(-19,2)	-1(-6,5)
model 5 = model 1+ mobility limitations	177(-279,633)	5(-9,18)	-6(-16,5)	1(-5,6)
model 6 = model 1+ fear of falling	270(-192,732)	3(-11,17)	-5(-16,6)	2(-4,7)
model 7 = model 1+ depression	46(-411,503)	8(-5,22)	-8(-18,3)	0(-6,5)
model 8 = model 1+ quality of life	338(-118,794)	2(-11,16)	-5(-16,6)	3(-3,8)
model 9 = model 1+ all	289(-125,703)	4(-9,16)	-6(-16,4)	2(-3,7)
**(b)No fall vs >=2 falls in past 12 months**	**Steps/day**	**Sedentary (minutes/day)**	**Light (minutes/day)**	**MVPA (minutes/day)**
model 1 = falls in past 12 months	-942(-1381,-503)	22(9,35)	-12(-22,-2)	-10(-15,-5)
model 2 = model 1+ exercise outcome expectations	-577(-995,-159)	12(-0,25)	-6(-16,4)	-6(-11,-1)
model 3 = model 1+ exercise self-efficacy	-345(-755,64)	8(-4,21)	-4(-14,5)	-4(-9,1)
model 4 = model 1+ number of days leave the house	-512(-941,-84)	10(-2,23)	-4(-14,6)	-6(-11,-1)
model 5 = model 1+ mobility limitations	-371(-814,71)	6(-7,19)	-1(-11,9)	-5(-10,1)
model 6 = model 1+ fear of falling	-299(-753,155)	6(-8,19)	-2(-13,8)	-3(-9,2)
model 7 = model 1+ depression	-659(-1092,-225)	13(1,26)	-6(-16,4)	-7(-12,-2)
model 8 = model 1+ quality of life	-275(-717,166)	7(-6,20)	-4(-14,6)	-3(-8,2)
model 9 = model 1+ all	293(-122,708)	-10(-23,2)	8(-2,18)	2(-3,7)

^1^coefficients from random effects regression models, accounting for clustering within person; wear days (range 3-7 days) at level 1 and all other variables at level 2, n = 1398.

Model 1 = falls+ minutes of accelerometer wear time + region of residence + day order + age +season of wear.

Model 9 = Model 1+exercise outcome expectations+ exercise self-efficacy+ number of days leave the house+ mobility limitations+ fear of falls+ depression+ quality of life.

### Fear of falling and physical activity

16% (n = 254/1577) of men were “very” or “somewhat” fearful of falling, they were more likely to have mobility difficulties, to leave the house less often, to have lower exercise self-efficacy and lower expectations about the benefits of exercise, lower quality of life and higher levels of depression, (Table 1). Men reporting FOF took significantly fewer steps per day; -1766(95% CI -1391, -2142) and spent more minutes in sedentary 45(95% 34, 56), and less in light -27(95% CI -18, -36) or MVPA -18(95% CI -13, -22), (Table 3, Model 1). These differences were very strongly mediated by lower exercise self-efficacy (Model 3). Other important mediators were higher levels of mobility limitations (Model 5), lower quality of life (Model 8), leaving the house on fewer days (Model 4), and exercise outcomes expectations (Model 2). Presence of depression (Model 7) and falls history (Model 6) changed the estimates a small amount. The differences in sedentary, light and MVPA were attenuated but remained significant on adjustment for each of the mediators in turn (Models 2–8). The differences in step count and minutes of sedentary, light and MVPA were fully mediated in Model 9 which included all the covariates.

**Table 3:** **Adjusted mean difference (95% CI) in PA between men fearful of falling vs not, (n = 1398)** ^**1**^

Fear of falling vs no fear of falling	Steps/day	Sedentary (minutes/day)	Light (minutes/day)	MVPA (minutes/day)
model 1 = fear of falling	-1766(-2142,-1391)	45(34,56)	-27(-36,-18)	-18(-22,-13)
model 2 = model 1+ exercise outcome expectations	-1280(-1647,-913)	32(21,43)	-19(-28,-11)	-13(-17,-8)
model 3 = model 1+ exercise self-efficacy	-846(-1219,-472)	24(13,36)	-16(-25,-7)	-8(-13,-4)
model 4 = model 1+ number of days leave the house	-1302(-1678,-926)	32(21,43)	-19(-28,-10)	-13(-18,-9)
model 5 = model 1+ mobility limitations	-1154(-1561,-746)	27(15,39)	-14(-24,-5)	-12(-17,-7)
model 6 = model 1+ history of falls	-1705(-2105,-1305)	43(31,55)	-26(-35,-17)	-17(-22,-12)
model 7 = model 1+ depression	-1434(-1814,-1053)	34(23,46)	-20(-29,-11)	-15(-19,-10)
model 8 = model 1+ quality of life	-1053(-1462,-644)	30(17,42)	-19(-29,-10)	-10(-15,-6)
model 9 = model 1+ all	-201(-607,205)	4(-8,17)	-2(-12,8)	-2(-7,3)

^1^coefficients from random effects regression models, accounting for clustering within person; wear days (range 3-7 days) at level 1 and all other variables at level 2.

Model 1 = fear of falling+ minutes of accelerometer wear time + region of residence + day order + age +season of wear.

Model 9 = Model 1+exercise outcome expectations+ exercise self-efficacy+ number of days leave the house+ mobility limitations+ falls+ depression+ quality of life.

### Fear of falling and physical activity- impact of having had a fall

16% (n = 254) of men reported FOF, of which 52% (n = 133) had fallen in the past year [15% (n = 37) one fall and 38% (n = 96) recurrent falls]. 41% (n = 133/325) of those who fell reported FOF. There was no evidence that FOF had a greater impact on PA levels among men who had fallen compared to those who had not fallen (LRT, p > 0.4 in each case).

As a sensitivity analysis, lower cut points of 25 cpm and 50 cpm (rather than 100 cpm) were used to define sedentary behaviour. Whilst these cut points identify fewer minutes of sedentary behaviour each day, the patterns of associations between falls or fear of falling and sedentary behaviour were unchanged.

## Discussion

In this large community-based sample, one in five older men fell in the past year, of whom half had recurrent falls, and one in six men reported FOF. In line with other studies, FOF was more common in, but not restricted to men with a history of falls [12]; only half of the men with FOF had actually fallen in the past year.

In our study, prevalence of falls was a little lower than other comparable studies [7, 11, 12, 15, 19], and FOF was less prevalent than in some other studies [9–11]. This could reflect selection bias, but even if our participants were more active and less fearful of falling than the average, any bias should underestimate true associations between falls or FOF and PA levels. Also, studies which ascertain falls using prospective monthly follow-up may report higher prevalences of falls than studies using a single item recall over the past year. Our FOF scale is a one item question with 3 possible answers. The scale has construct validity: in line with expectations from other studies, men with FOF had lower quality of life and more mobility limitations than those who were not fearful [31]. A variety of single item questions have been used in many other studies to identify fear of falls [14]. A similar single item question is reported to correlate well with validated scales including the Falls Efficacy Scale (r = 0.43) and the Survey of Activities and Fear of Falling in the elderly scale (r = -0.59) [31].

Levels of objectively measured PA were similar in men who did not fall and men who fell once. However, compared to non-fallers, recurrent fallers spent on average 20 minutes more per day in sedentary behaviours. They spent less time in all domains of PA; 942 fewer steps and 12 minutes less light activity and 10 minutes less MVPA per day, suggesting that both total volume and intensity of activity are reduced. The findings quantifying how the different intensities of PA and sedentary behaviour relate to falls history are novel, but they fit with reports that self-reported PA is curtailed after a fall [7, 19] and that inactivity may lead to falls. Findings suggest that recurrent fallers have different characteristics to single fallers.

The deficits in total volume and in intensity of PA seen among men with FOF were approximately twice as large as the deficits seen among recurrent fallers compared to non-fallers. FOF was associated with 1766 fewer steps/day and 27 minutes less of light activity, 18 minutes less of MVPA along with an increase of 45 minutes/day in sedentary behaviour. Whilst the deficits in MVPA were small in absolute terms (18 minutes/day), because total MVPA levels are very low in older adults (38 minutes/day in our sample), and decline with advancing age [1], in relative terms, this was a large decrease. The deficit has potentially detrimental consequences, including loss of strength and balance, which may be important in eventual loss of independent living [17]. Although many studies have established that fear of falling is associated with activity limitations [11–15], it is hard to compare the size of associations from previous studies of total self-reported PA directly with our measures of daily minutes in different intensities of PA.

We examined several potential mediators which might account for differences in PA between fallers and non-fallers and men with and without FOF. Mediators were chosen if they had been reported to be associated with PA levels and with falls or FOF in other studies, and were also related to falls, FOF and PA levels in our study. We found that lower quality of life, lower exercise self-efficacy, leaving the house on fewer days, and, to a lesser extent, mobility difficulties outdoors and lower expectations of benefits of exercise were potential mediators between falls or FOF and activity levels. Indeed these mediators were mostly consistent across all the domains of activity (daily step counts and minutes of sedentary, light and MVPA). The same mediators were also important in mediating between both falls history and PA outcomes and also between FOF and PA outcomes, not surprising given that falls history and FOF are closely inter-related. Exercise self-efficacy and outcome expectation are important constructs from the domain of theory of behaviour change and in our study, as elsewhere, were strongly predictive of activity levels [25]. The number of journeys outside the home may importantly determine PA levels in older adults [21] and a fewer journeys outside the home may indicate activity limitation. Some activity limitation will be due to limitations in indoor activity, however we did not ask specific questions about indoor activities, so cannot specifically investigate this. There was little evidence that presence of depression, indexed by the GDS, was a strong mediator, perhaps because our measure was limited or because other measures of mental health status (including anxiety) might be more relevant. FOF was a strong mediator between recurrent falls and all aspects of activity levels. However associations between FOF and activity were little affected by adjustment for falls history. Additionally we did not find evidence that the associations between FOF and activity levels were modified by falls history, suggesting that the FOF-PA association is independent of actual falls history. Although FOF and recurrent falls overlap, there seems to be a distinct and stronger association between fear of falling and PA than with falls history.

### Strengths and limitations

This study extends literature about how falls and FOF are associated with PA in several ways. Firstly, we use objectively measured PA which, unlike self-reports, is not susceptible to recall or reporting bias. Whilst other studies have established that FOF is associated with limitations in self-reported PA [11–13, 15, 16], the impact on different intensities of activities and on sedentary time have not been studied. Secondly, we accounted for important confounding factors. Thirdly, we investigated a wide range of mediators to understand the activity deficits in recurrent fallers and those with FOF. Fourthly, our sample is large and includes ambulatory community-dwelling men, not an “at risk” population, (eg adults in residential care), so results are widely generalizable. However the cross-sectional study design prevents us from identifying the direction of causality, which may be bidirectional [32]. Our sample is limited to men so our findings cannot be extrapolated to women. We know from previous research that the prevalence of falls is higher among women than men [32], that women are more likely to report fear of falling than men [14], that women are more likely to inappropriately perceive themselves to be at high risk of falls than men [33] and that women have lower MVPA and higher levels of sedentary behaviour than men [34]. It is therefore important that future studies explore whether the relationship between PA, FOF and falls varies by gender. The accelerometer data permitted us to estimate the size of the changes in time spent in sedentary, light and MVPA associated with falls or FOF. Whilst the coefficients for sedentary, light activity and MPVA must balance out and sum to zero, we have independent data on two of the three outcomes. It is nevertheless a strength to investigate associations with objectively measured time spent in different intensities of activity and with step counts, as these data give us insights that questionnaire data would not give us. It is possible that there is some reactivity to wearing an accelerometer, ie becoming more active, we found that there was a small excess on the first day of accelerometer wear and estimates from subsequent days were very consistent. Indeed, we specifically accounted for this issue in our analyses by controlling for day order in statistical models. We note that there are no universally agreed accelerometer cut-points to define sedentary behaviour in older adults, we used the most commonly used definition of 100 cpm, [35] which permits our results to be compared to other data. However sensitivity analyses using recently suggested lower cut-points of 25 cpm [28] and 50 cpm [29], did not alter our conclusions about associations between falls or fear of falling and sedentary behaviour.

## Conclusions

Fear of falls and actual falls are important barriers to older people benefitting from walking and MVPA, and may promote sedentary behaviour, with its own detrimental health effects. The strong association between FOF and lower step counts and time spent in light and MVPA may have serious adverse consequences for the many outcomes associated with lower PA levels [4], including future risk of falls. FOF may reduce activity levels through reduced exercise self-efficacy and fewer journeys outside the home which, if replicated in longitudinal studies, may be areas for intervention to reduce the activity-limiting effects of FOF. However, as FOF may protect against falls, interventions to increase PA levels in adults with FOF also need to reduce falls risk, otherwise targeting FOF could actually increase falls.

## Abbreviations

CI: Confidence interval
CPM: Counts per minute
FOF: Fear of falling
GDS: Geriatric depression score
LRT: Likelihood ratio test
MVPA: Moderate to vigorous physical activity
PA: Physical activity
SD: Standard deviation.
